# Supplementary material for: Dyslipidaemia Is Associated with Severe Disease Activity and Poor Prognosis in Ulcerative Colitis: A Retrospective Cohort Study in China
Source: Nutrients. 2022 Jul 24;14(15):3040. doi: 10.3390/nu14153040 (PMC9330762; doi:10.3390/nu14153040)
Supplement: Supplementary file 1 [file nutrients-14-03040-s001.zip › nutrients-1795059-supplementary.pdf]

Supplementary Materials

Supplementary Table S1. Comparison of clinical features in the patients with biological remission and endoscopic remission

Variables (%), (*n*=328)	Biological remission (*n*=270)	*p* value	Endoscopic remission (*n*=164)	*p* value
Sex (male)	142 (52.6)	0.55	82 (50.0)	0.51
[a] Disease duration	137 (50.7)	0.73	86 (52.4)	0.44
BMI ≥ 23 (kg/m^2)	95 (35.2)	0.00**	66 (40.2)	0.57
EIMs	32 (76.2)	0.27	22 (13.4)	0.74
Persistent dyslipidaemia	51 (18.9)	0.36	18 (11.0)	0.00**
Medications				
Aspirin	9 (3.3)	1.00 [c]	6 (3.7)	0.75 [b]
5-ASA	250 (92.6)	0.03*c	153 (93.3)	0.64
Glucocorticoids	210 (77.8)	0.01*b	124 (75.6)	0.03*
IM	89 (33.0)	0.22	55 (33.5)	0.73
Biological therapy	42 (15.6)	0.52	24 (14.6)	0.45

Statistics are performed using linear regression for continuous variables and Chi-squared test for categorical variables. When appropriate, values are reported as median [IQR] or number (%). IQR= interquartile range. [a] Disease duration: time from onset to follow up ≥ 10 years. BMI = Body Mass Index. EIMs=Extraintestinal manifestations. 5-ASA=5-Aminosalicylic Acid. IM= Immunosuppressant. [b] Continuity correction. [c] Fisher's test. Significant *p*-value < 0.05. $^{*}p < 0.05$, $^{**}p < 0.01$, $^{***}p < 0.001$.

Supplementary Table S2. Logistic regression analysis in the patients with biological remission and endoscopic remission

Variables	Biological remission (*n*=270)			Endoscopic remission (*n*=164)		
	p value	OR	95%CI	*p* value	OR	95%CI
Sex (male)	0.40	1.30	0.70-2.38	0.43	0.83	0.52-1.32

[a] Disease duration	0.47	1.25	0.68-1.27	0.23	1.33	0.84-2.08
BMI ≥ 23（kg/m^2）	0.006**	0.43	0.23-0.78	0.29	0.77	0.81-2.08
EIMs	0.80	0.69	0.31-1.54	0.87	1.05	0.53-2.04
Persistent dyslipidaemia	0.72	0.69	0.30-1.62	0.00**	0.34	0.18-0.64
Medications						
Aspirin	0.71	1.52	0.17-12.50	0.75	1.25	1.56-5.56
5-ASA	1.00	0.01	0.00-	0.68	0.81	0.29-2.22
Glucocorticoids	0.046*	0.33	0.11-0.98	0.039*	0.52	0.28-0.96
IM	0.60	0.84	0.44-1.59	0.68	1.11	0.67-1.85
Biological therapy	0.84	1.09	0.49-2.44	0.45	0.78	0.40-1.49

[a] Disease duration: time from onset to follow up ≥ 10 years. BMI = Body Mass Index. EIMs=Extraintestinal manifestations. 5-ASA=5-Aminosalicylic Acid. IM= Immunosuppressant. OR=Odds Ratio. 95% CI = 95% confidence interval. Significant *p*-value < 0.05. $^{*}p < 0.05$, $^{**}p < 0.01$.

Supplementary Table S3. Univariate and multivariate Cox regression analysis of risk factors for surgery and tumorigenesis

Variables	Univariate analysis						Multivariate analysis					
	Surgery (*n*=83)			Tumorigenesis (*n*=40)			Surgery (*n*=83)			Tumorigenesis (*n*=40)		
	p value	HR	95%CI	*p* value	HR	95%CI	*p* value	HR	95%CI	*p* value	HR	95%CI
Sex (male)	0.02*	1.69	1.08-2.64	0.25	1.46	0.77-2.76	0.08	1.50	0.96-2.36	—	—	—
Age at onset	0.00**	1.02	1.00-1.04	0.05	1.03	1.00-1.05	0.75	1.02	1.00-1.03	0.33	1.01	0.99-1.03
[a] Disease duration	0.00***	3.18	0.18-0.57	0.11	0.40	0.13-1.22	0.00***	0.31	0.17-0.56	0.04*	0.29	0.09-0.95

BMI ≥ 23 (kg/m²)	0.36	0.82	0.53-1.26	0.43	0.88	0.64-1.21	—	—	—	—	—	—
EIMs	0.87	0.95	0.49-1.84	0.93	0.18	0.03-1.33	—	—	—	—	—	—
Persistent dyslipidaemia	0.00***	3.15	1.81-5.49	0.00***	7.46	3.79-14.69	0.00***	3.27	1.86-5.75	0.00***	7.92	3.97-15.78
Aspirin	0.34	0.51	0.12-2.07	0.48	0.49	0.07-3.58	—	—	—	—	—	—
5-ASA	0.34	1.64	0.59-4.54	0.15	0.52	0.21-1.28	—	—	—	—	—	—
Glucocorticoids	0.88	1.83	0.91-3.65	0.68	0.85	0.39-1.85	—	—	—	—	—	—
IM	0.08	1.48	0.95-2.31	0.51	0.78	0.38-1.62	—	—	—	—	—	—
Biological therapy	0.38	0.73	0.37-1.47	0.84	0.17	0.02-1.27	—	—	—	0.06	0.15	0.02-1.10

[a] Disease duration: time from onset to follow up ≥ 10 years. BMI = Body Mass Index. EIMs=Extraintestinal manifestations. 5-ASA=5-Aminosalicylic Acid. IM= Immunosuppressant. HR=Hazard Ratio. 95% CI = 95% confidence interval. Significant *p*-value < 0.05. $^{*}p < 0.05$, $^{**}p < 0.01$, $^{***}p < 0.001$.

Supplementary Table S4. Comparison of lipid profiles in the patients with surgery or tumorigenesis

Lipid levels median [IQR]	Surgery (n=328)		p value	Tumor (n=328)		p value
	With surgery (n=83)	Surgery-free (n=245)		Tumor-bearing (n=40)	Tumor-free (n=288)	
Lipid levels						
Normal lipid level, n (%)	18 (21.7)	182 (74.3)		4 (10.0)	196 (68.1)	
Transient dyslipidaemia, n (%)	20 (24.1)	49 (20.0)	0.00***	2 (5.0)	67 (23.2)	0.00***
Persistent dyslipidaemia, n (%)	45 (54.2)	14 (5.7)		34 (85.0)	25 (8.7)	
Laboratory findings						
TC (mmol/l)	4.2 [3.1-5.2]	4.6 [4.0-5.2]	0.01*	4.8 [3.7-5.6]	4.5 [3.9-5.2]	0.39
TG (mmol/l)	1.3 [1.0-2.0]	1.2 [0.9-1.6]	0.01*	1.2 [1.0-2.3]	1.2 [0.9-1.7]	0.28
HDL-C (mmol/l)	0.9 [0.7-1.2]	1.2 [1.1-1.5]	0.00***	0.9 [0.8-1.1]	1.2 [1.0-1.4]	0.00***
LDL-C (mmol/l)	2.3 [1.8-3.1]	2.7 [2.2-3.2]	0.04*	2.8 [2.1-3.6]	2.6 [2.1-3.1]	0.16
ApoA1 (g/l)	1.1 [1.0-1.2]	1.4 [1.2-1.6]	0.00***	1.1 [1.0-1.2]	1.3 [1.2-1.6]	0.03*
ApoB (mg/l)	0.8 [0.7-1.1]	0.8 [0.7-1.0]	0.75	0.9 [0.7-1.0]	0.8 [0.7-1.0]	0.97
Lp α (mg/l)	146.0 [47.0-338.0]	109.0 [52.5-211.0]	0.49	262.5 [41.3-368.0]	108.0 [54.8-224.5]	0.30
FFA (umol/l)	539.0 [334.0-608.0]	456.0 [341.0-723.5]	0.95	587.5 [449.0-681.5]	476.0 [334.0-640.0]	0.36
hsCRP (mg/l)	18.0 [3.4-58.5]	3.0 [0.8-11.2]	0.00***	7.0 [2.9-52.0]	3.6 [0.9-19.0]	0.02*

Statistics are performed using linear regression for continuous variables and Chi-squared test for categorical variables. When appropriate, values are reported as median [IQR] or number (%). IQR= interquartile range. TC=Total cholesterol. TG=Total triglyceride. HDL-C=High-density lipoprotein cholesterol. LDL-C=Low-density lipoprotein cholesterol. ApoA1=Apolipoprotein A1. ApoB=Apolipoprotein B. Lp α=Lipoprotein α. FFA=Free fatty acid. hsCRP=Hypersensitivity C-reaction protein. Significant p-value < 0.05. $^{*}p<0.05$, $^{***}p < 0.001$.

Supplementary Table S5. Correlation analysis between lipid profiles and hsCRP levels

	Items	TC	TG	HDL-C	LDL-C	ApoA1	ApoB	Lpα	FFA
hsCRP	Pearson correlation	-0.08	0.132	-0.39	-0.04	-0.48	0.038	0.006	0.038
	p value	0.18	0.05	0.00**	0.54	0.00**	0.79	0.97	0.78

Pearson correlation analysis was performed between lipid profiles and hsCRP levels in UC patients with multi-points of lipid measurements. hsCRP=Hypersensitivity C-reaction protein. TC=Total cholesterol. TG=Total triglyceride. HDL-C=High-density lipoprotein cholesterol. LDL-C=Low-density lipoprotein cholesterol. ApoA1=Apolipoprotein A1. ApoB=Apolipoprotein B. Lp α=Lipoprotein α. FFA=Free fatty acid. Significant p-value < 0.05. $^{**}p < 0.01$.
